# Categorizing SHR and WKY rats by chi2 algorithm and decision tree

**DOI:** 10.1038/s41598-021-82864-3

**Published:** 2021-02-10

**Authors:** Ping-Rui Tsai, Kun-Huang Chen, Tzay-Ming Hong, Fu-Nien Wang, Teng-Yi Huang

**Affiliations:** 1 Department of Physics, National Tsing Hua University, Hsinchu, 30013 Taiwan, ROC; 2 Present Address: College of Management and Design, Ming Chi University of Technology, New Taipei City, 243303 Taiwan, ROC; 3 Department of Biomedical Engineering and Environmental Sciences, National Tsing Hua University, Hsinchu, 30013 Taiwan, ROC; 4 Department of Electrical Engineering, National Taiwan University of Science and Technology, Taipei, 10607 Taiwan, ROC

**Keywords:** Computational biology and bioinformatics, Neuroscience, Diseases

## Abstract

Classifying mental disorder is a big issue in psychology in recent years. This article focuses on offering a relation between decision tree and encoding of fMRI that can simplify the analysis of different mental disorders and has a high ROC over 0.9. Here we encode fMRI information to the power-law distribution with integer elements by the graph theory in which the network is characterized by degrees that measure the number of effective links exceeding the threshold of Pearson correlation among voxels. When the degrees are ranked from low to high, the network equation can be fit by the power-law distribution. Here we use the mentally disordered SHR and WKY rats as samples and employ decision tree from chi2 algorithm to classify different states of mental disorder. This method not only provides the decision tree and encoding, but also enables the construction of a transformation matrix that is capable of connecting different metal disorders. Although the latter attempt is still in its fancy, it may have a contribution to unraveling the mystery of psychological processes.

## Introduction

**Figure 1:**
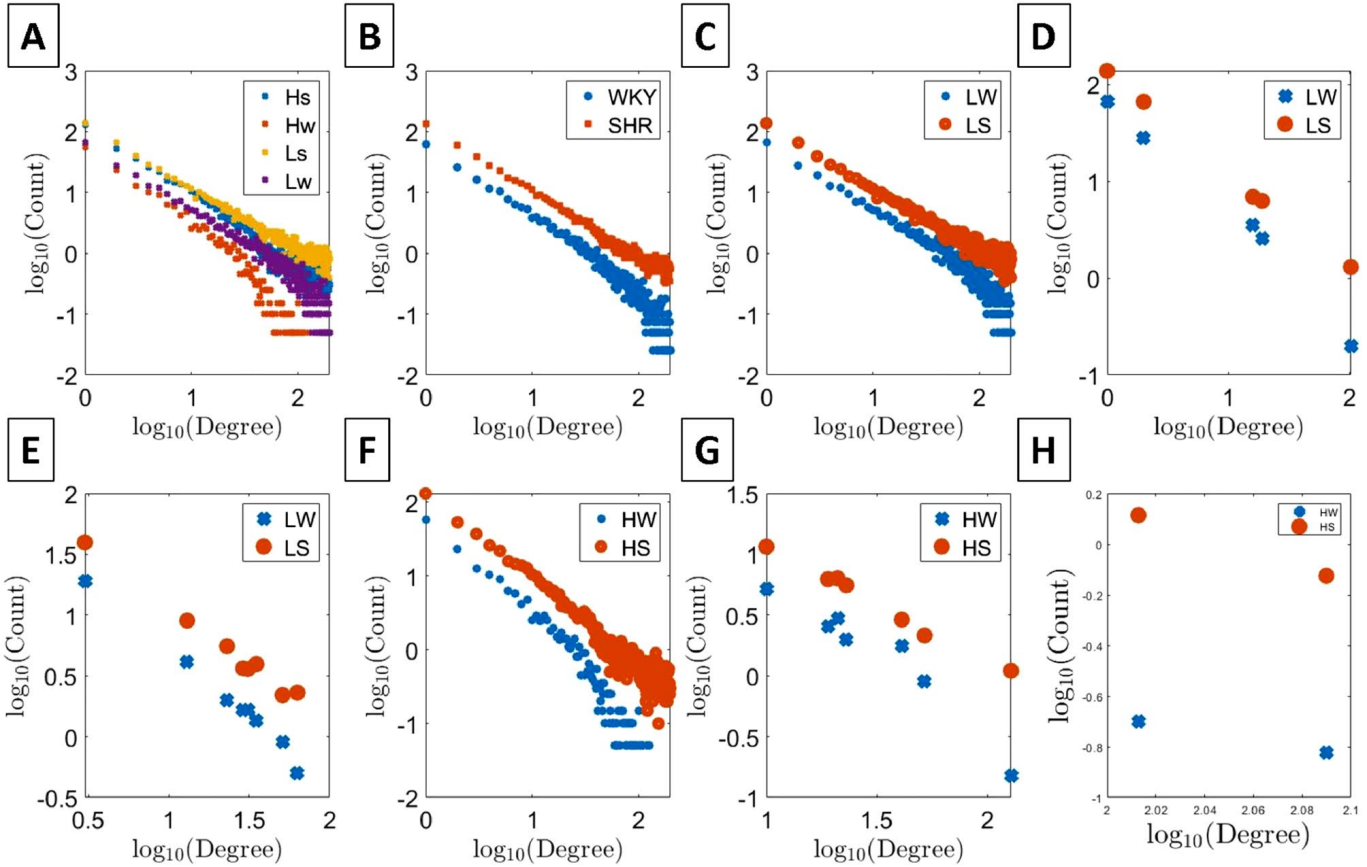
Power-law distribution consists of degree and its counted number. Each state, such as HS, HW, LS and LW, has 20 samples. Plots (**A**), (**B**), (**C**) and (**F**) are the distribution before chi2 algorithm. In (**B**), WKY and SHR are plotted without considering the anesthesia factor, and each distribution is deduced from 40 samples. In contrast, plots (**D**), (**E**), (**G**), and (**H**) are the distribution after chi2 algorithm.

What are the roots of mental disorder^1^ In the path to answering this question, researchers are beginning to untangle the common biology that links supposedly distinct psychiatric conditions. Along this line of efforts, the purpose of this study is to determine whether the combination of power law and decision trees would improve the efficiency at classifying different mental disorders or states. In light of such a concern, this article comprises two steps: (a) providing a method to encode fMRI to the power-law distribution; (b) using decision trees to classify encoding to correct state. Recent studies showed that human brain activity can be expressed by different network equations^2^ with the aid of graph method by fMRI samples^3^. Power law has been reported for many complex physical systems. Examples are the city population^4^, world wide web^5^, fluctuations in financial market^6^ et al. In this paper, fMRI information are encoded by power law distribution. we discuss the power law trait of authorized^7^ rat samples with SHR and WKY. Besides, Isoflurane (ISO) is used to further divide our samples into four states: high isoflurane WKY = HW, high isoflurane SHR = HS, low isoflurane SHR = LS, and low isoflurane WKY = LW. The format of our sample is 11 slices, 525 times, $64 \times 64$, FOV = 30 mm, and slice thickness = 1 mm. And each state contains 20 data. mental disorder is regarded as the mental difference, isoflurane is regarded as stimuli.

We use the same procedure as Ref.^1^ to obtain the power-law network distribution for the fMRI data of rats. Pearson correlation defined in Eq. (1) plays an important role because this calculable quantity can reflect the strength of positive correlation between any two voxels:

1 $$\begin{aligned} r(x_1, x_2)=[\overline{v(x_1,t)v(x_2,t)}-\overline{v(x_1,t)}\times \overline{v(x_2,t)}]/[\sigma (x_1)\sigma (x_2)]\ \end{aligned}$$

where a voxel at position *x* and time *t* is denoted as *v*(*x*, *t*) while $\sigma$ represents the standard deviation. When Eq. (1) exceeds a threshold value, chosen to be 0.7, these two voxels are regarded as being linked. After statistically analyzing all different degree of connectivity in the whole brain, we can obtain the power law distribution. Figure 1A shows the average distribution of these four states and Fig. 1B,C and F are average distribution of two states. Power law distribution is extremely useful because it sheds light on the difficult problem of analysing mental states. But it is insufficient to merely use its exponent to distinguish samples because ROC index is always less than 0.7. Based on this reason, chi2 algorithm will be quoted to help us select the significant difference from a group of degrees.

**Figure 2:**
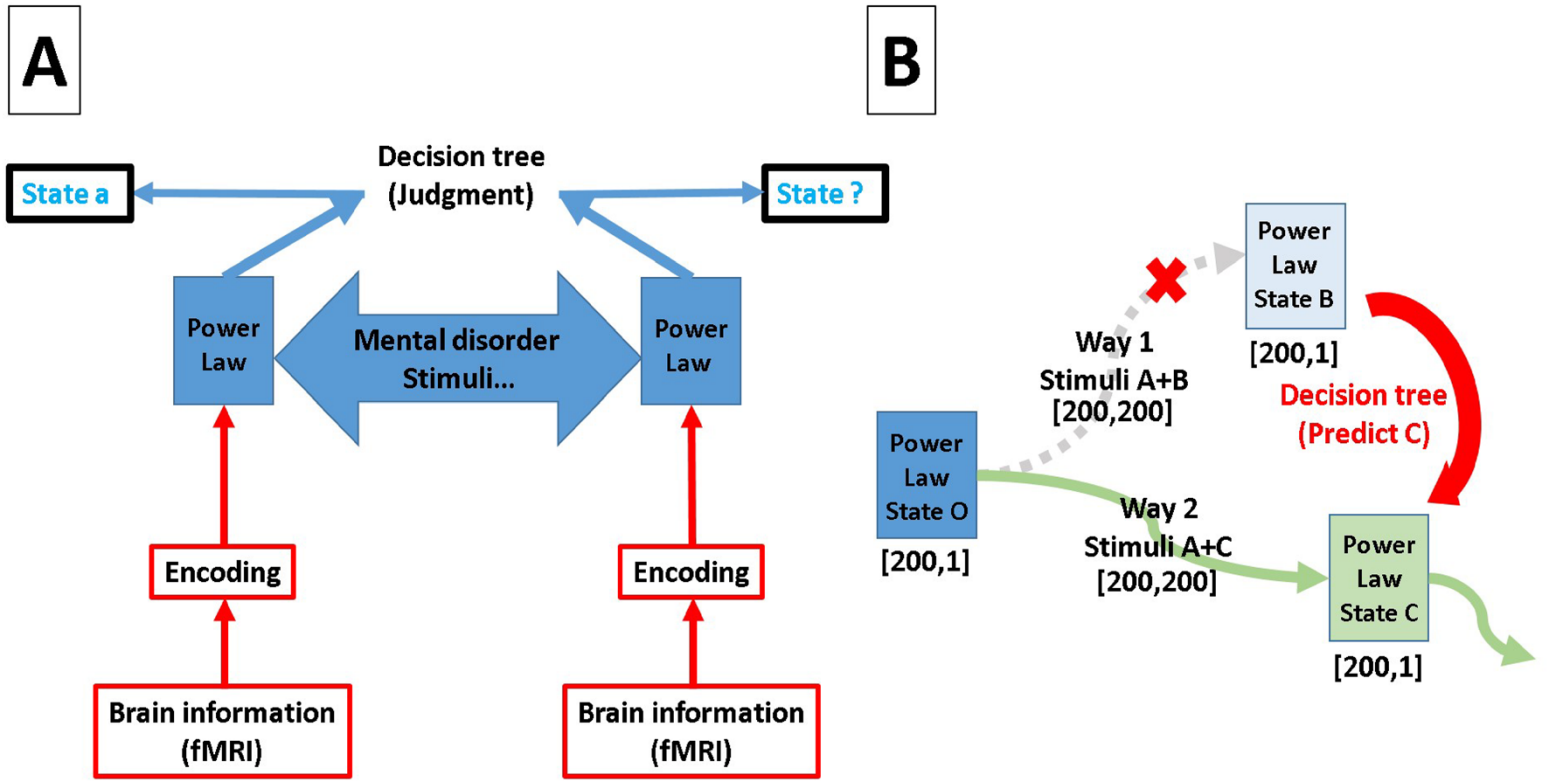
Plot (**A**) is a flow chart that includes encoding fMRI to power law and using decision tree to classify states. Plot (**B**) shows the transition between two states. If a situation has several paths, decision tree can help us detect which is the right way during evolution.

After chi2 algorithm.C4.5 decision trees can produce a tree structure. Chi2 algorithm is one of them of algorithm to make tree, ID3 and ID5 and so on is popular too, Fig. 1D,E,G and H are distribution made by chi2 algorithm. There are significant differences between groups. In order to test ROC of tree. We use tenfold validation to avoid over-fitting. Our purpose is to demonstrate whether C4.5 decision trees can offer a better way to help us observe and detect power law. Decision trees is a very popular tool for classification in data mining, which is widely used in deep-learning and machine learning^8,9^, industrial application^10,11^, medical treatments^12–14^, and bioinformatics^15–17^. To familiarize the readers with how decision trees can be of use in practical problems, let’s imagine if we want to know who was dead among the passengers who boarded the Titanic. First, we can quote the list of passenger, such as gender, age or level of class on the boat. Second, using this list to make decision trees. Finally, decision trees will tell us which condition can effect the fate of each passenger. Obviously, in this paper power law is the analogy of the list - different degrees are like the factors. In the Result section of this paper, we will ensure the relation between C4.5 decision trees and the encoding fMRI of power law, such as a bar code and detector. This relation is rule how to readout sample condition, see Fig. 2A. In addition to this, this relation can help us to face a psychological processing which Encoding will be changed during dynamical, see Fig. 2B. in this project, we use MATLAB to deal with fMRI raw data. The GPU and CPU mixing program allows us to increase the execution efficiency. The former transforms the 4D (3D voxel space and time) into a 2D matrix (voxel position and time), while the latter handles the calculation of correlation. As for the decision trees, we save the MATLAB matrix file by csv format, input the data into Excel, and output to Java to build the decision trees for the final calculation of the outcome of tenfold cross-validation. We construct the transform matrix (TM) by using linear regression to find the relation between two mental disorder states . For example, to obtain the TM from LS to LW, we treat LW as a target state and a dependent variable which is defined by averaging power law distributions of LW. Our data set consists of 15 power law distributions from LS, defined as the original state. The weights of 200 linear regression equations are then utilized to construct TM, as shown in Fig. 4A. The process of encoding and decision tree is arranged in the “Materials and methods” section.

## Results

**Figure 3:**
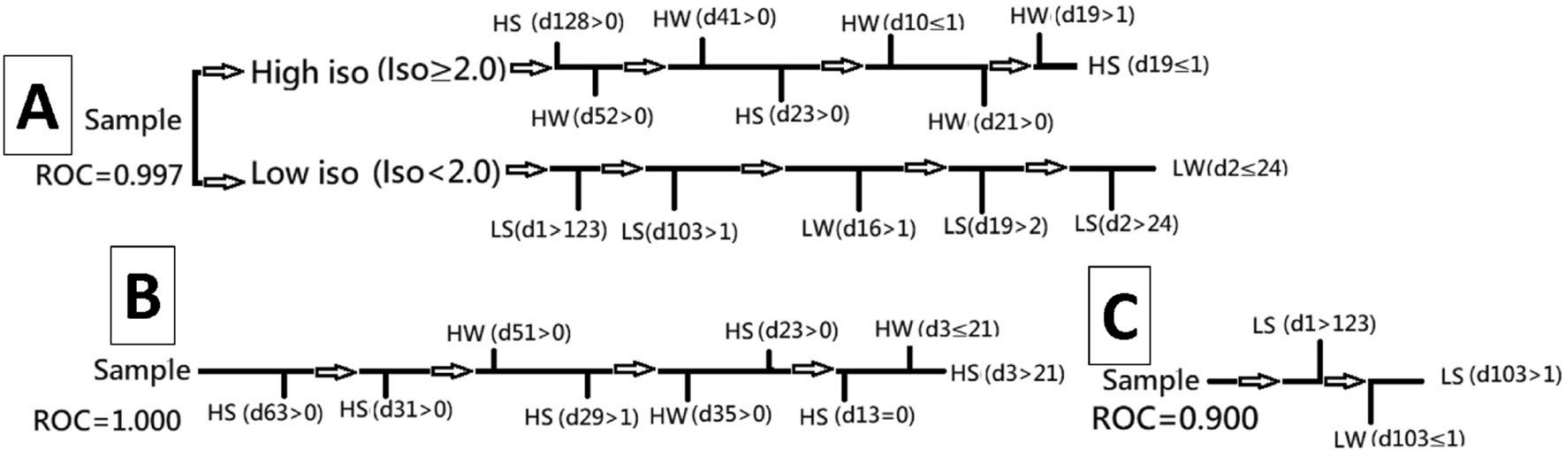
Plot (**A**) shows the decision tree of HS, HW, LS, and LW states. In contrast, plot (**B**) is of HS and HW states, and plot (**C**) of LS and LW states.

All the branches of decision tree are important features from chi2 algorithm, see Fig. 3, while Fig. 1D–H show the distribution for degree after chi2 algorithm. By comparing with Fig. 1A–C, one can see that Fig. 1D–H manage to widen the separation between distributions for degree. Now we can input important features into C4.5 by tenfold cross-validation before selecting the trees whose ROC approaches 0.9. Figure 3A explains how the four states are distinguished. Initially, data are categorized into two groups, based on whether the dosage of isoflurane (iso) $\ge 2.0$. Figure 3B and C show results from different situations of high ios and low ios groups. Our data are processed by ten fold cross-validation to achieve good results with $\mathrm{ROC}>0.8$. We double-confirm that power law has high sensitivity to represent the rat problem.

**Figure 4:**
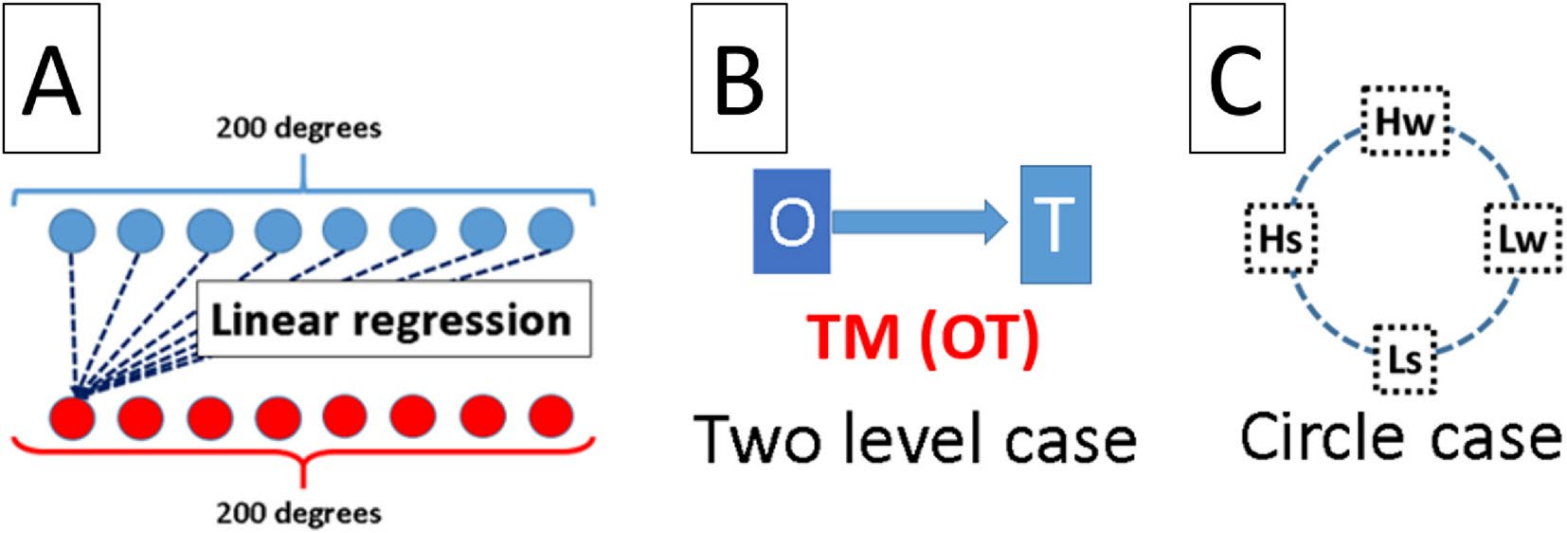
Plot (**A**) expresses TM constructed from linear regression between degrees. Plots (**B**) and (**C**) show the simulation about two level transition and four level transition.

**Figure 5:**
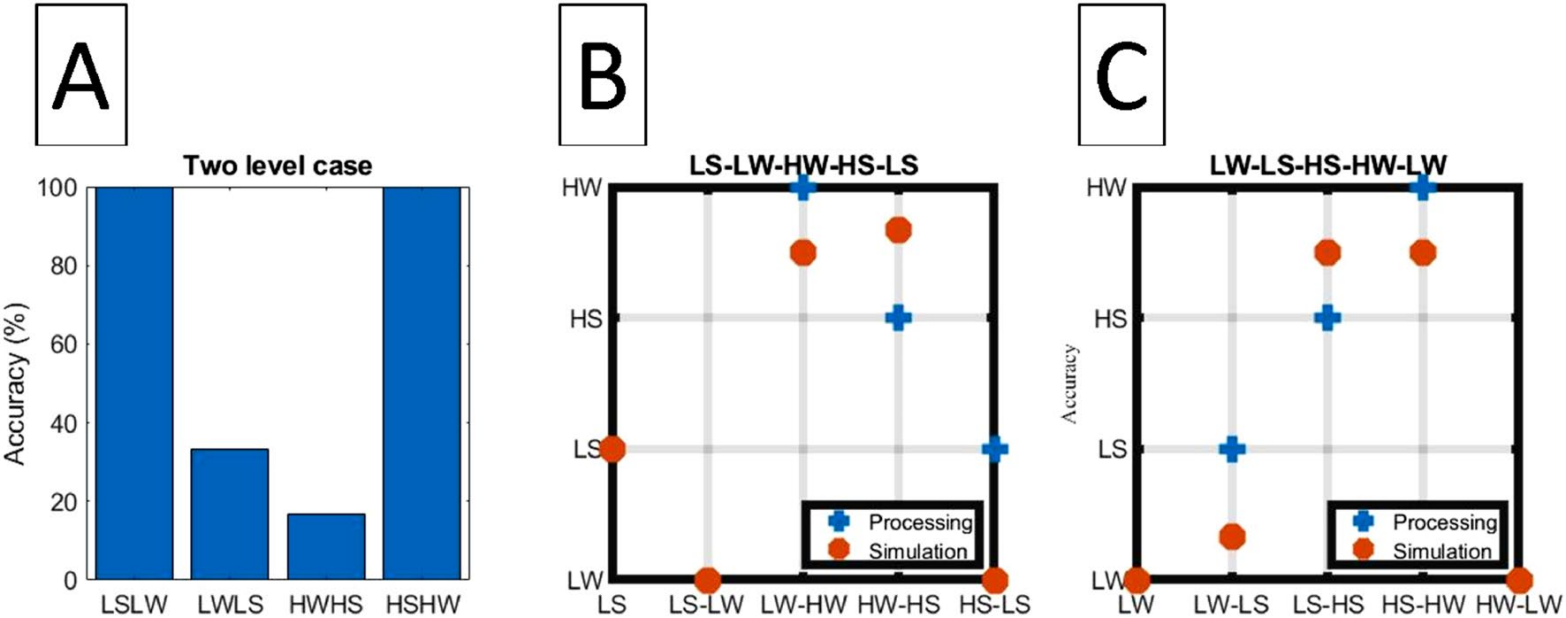
Plot (**A**) shows the accuracy in the case of two level transition from 5 testing samples. Compare with LSLW and HSHW that have a complete transition after detection by decision tree, the accuracy for LWLS and HWHS is lower. Several factors must be solved to obtain TM. Plots (**B**) and (**C**) show the circle case testing in Fig. 4B.

Schematic plot in Fig. 4B describes the relation between original and target states which we regard as a two-level case. After the transformation via TM and being judged by decision tree, the accuracy is shown in Fig. 5A. Figure 4C describes that four TM are applied for each original state in order to obtain the results in Fig. 5B and C.

## Discussion

**Figure 6:**
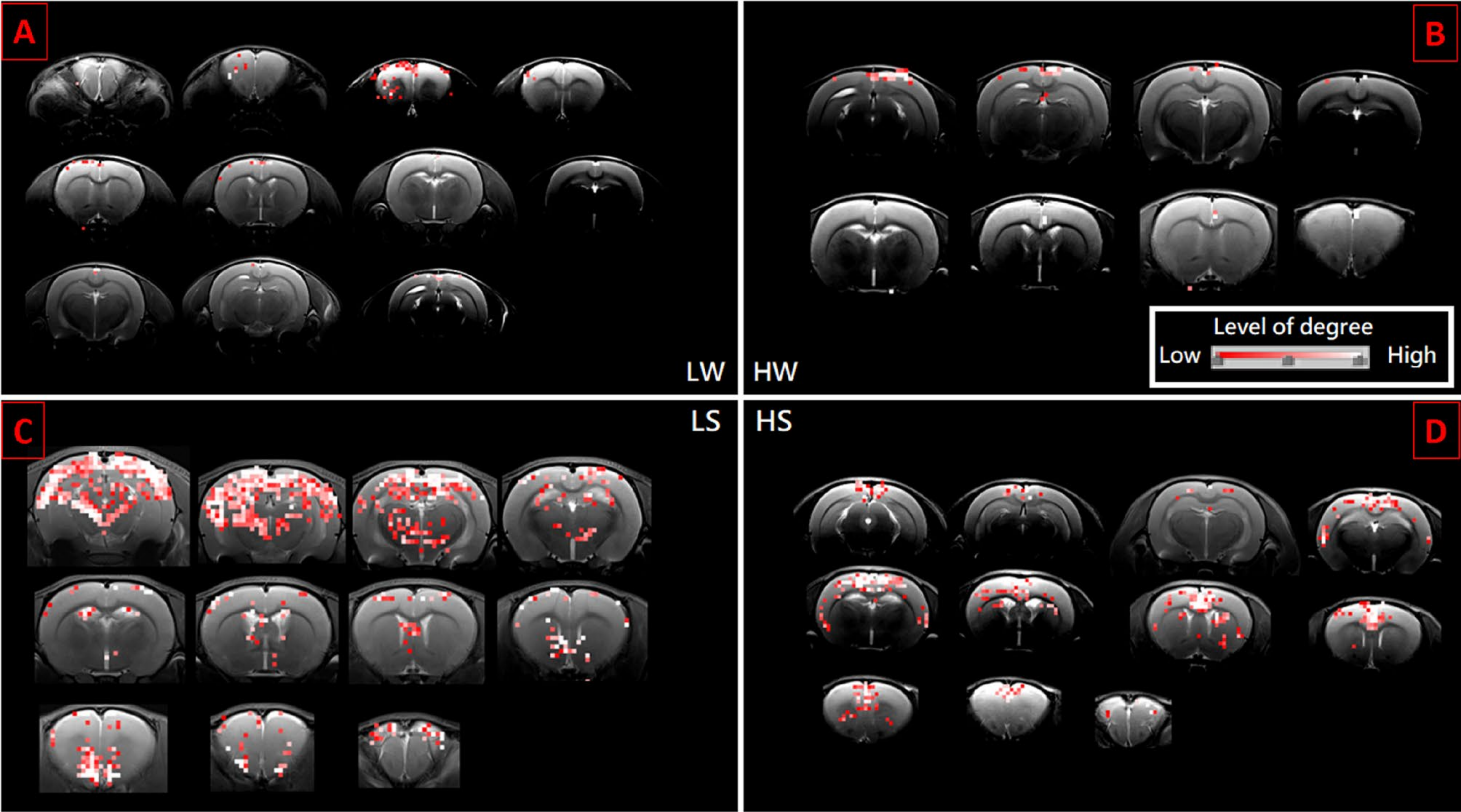
Panels (**A**) to (**D**) illustrate the activated region in LW, HW, LS, and HS states, respectively. The grade of brightness signifies different degrees of connectivity in the cross section. We only show the brain section with points.

**Figure 7:**
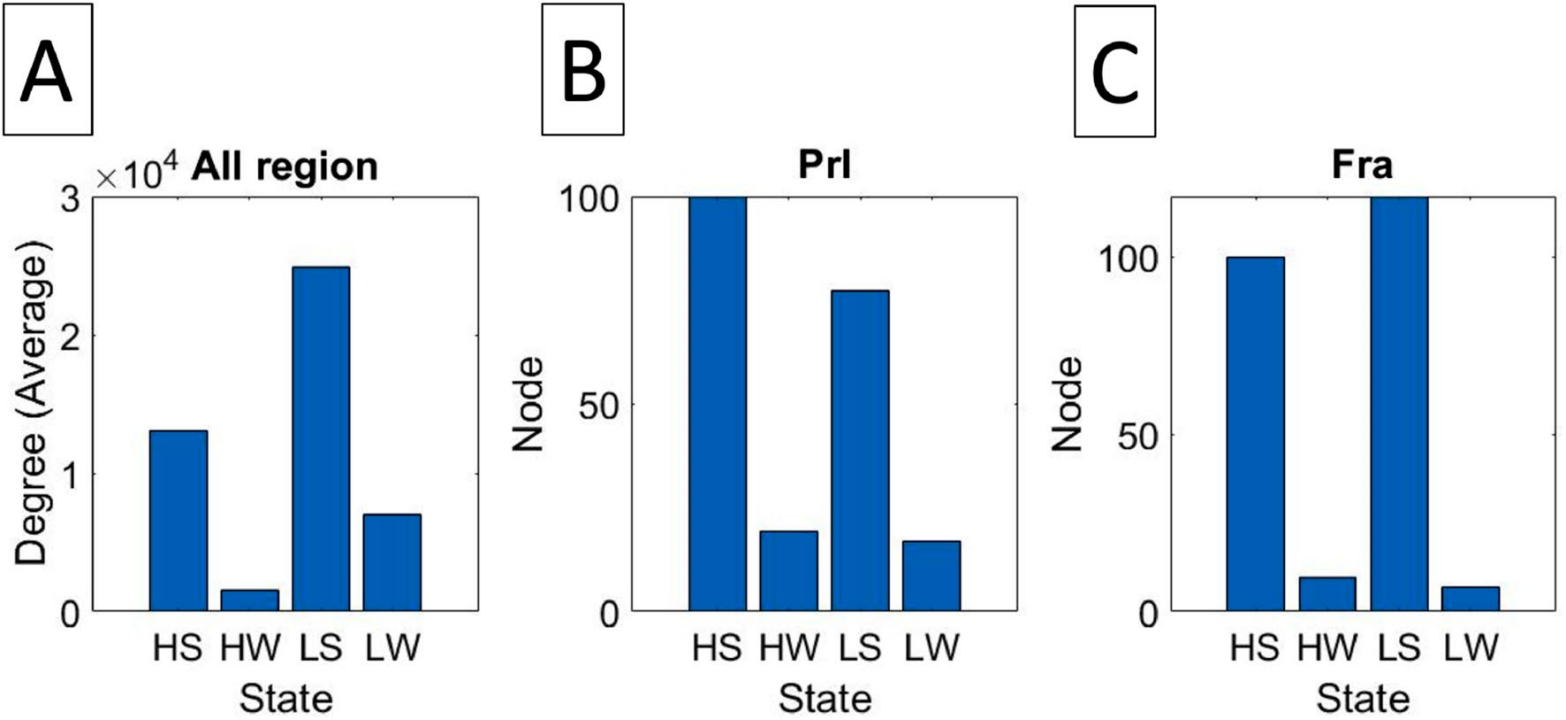
Plot (**A**) shows the total count of degree for the whole brain. Each state consists of 20 samples. (**B**) and (**C**) show Prl and Fra regions where the difference between SHR and WKY is significant. SHR has more nodes than WKY. Each state has 6 samples.

Figure 6 shows that all states have different distributions for degree. Only the most representative result is selected among 20 rat samples for each state. In general, SHR/low-ISO has more degrees and covers more brain regions than WKY/ high-ISO. The rank 1 in HS is the Primary motor cortex, while HW, LS, and LW the Secondary motor cortex. In the mean time, the rank 2 in HS is Retrosplenial agranular cortex, while HW, LS, and LW are Primary motor cortex. Whenever the brain develops disorder, it exhibits a different functional network that consequently gives rise to a new exponent for the power law in Fig. 1. This is similar to the finding of Ref.^2^ that the exponent may vary as the trial subject engages in different activities. We can find that the most active brain regions are the same. However, if we just focus on the samples of SHR, we discover that the secondary motor^18^ will fall to rank four. This is the reason why LS rat is more active than HS rat. More details will rely on more biological experiments in the future. The prefrontal cortex of ADHD patients has been reported to show abnormalities^19,20^. In our case, we can check two important regions in the prefrontal cortex to distinguish different states. These regions, Prl^21–24^ and Fra^25^, are related to the self-control and ADHD. The prefrontal cortex of SHR rat has been studied^26–28^. Figure 7A shows the summation of degree over whole brain region with 6 samples in 4 states, while Fig. 7B and C show the average number of node in Prl and Fra. Note that the stimulus interaction for LS being lower than LW in Fra is contrary to our expectation. Future biological experiments are needed to clarify the source of this problem.

One common method in RS-fMRI (Resting state-fMRI) is Seed-based Correlation Analysis (SCA). SHR and WKY have been studied^7^. In general, SCA requires the choice of a Region Of Interest (ROI) to be a priori assumption, and needs to average over seed regions before calculating connectivity. In contrast, we forgo this step to avoid any subjective and abnormal seed from affecting the outcome. Instead, we upgrade and simplify the graph theory by numerical representations. Whether this approach is applicable to all states of mental disorder and has restrictions are important questions to answer in the future. In this work we have managed to establish that the power-law distribution carries enough information to deal with the trial subjects in this case. To locate the characteristics of any mental state, one only needs to use chi2 algorithm to pick out important degrees filtered from the power-law distribution.

In the past, researchers found that brains have two large imposing systems in the resting state. One is the DMN (Default mode network), while the other is composed of attentional or task-based systems^29^. This motivates us to check whether double power laws may turn out to describe the distribution for degree better than the usual simple power law. In other words, can it be that each of these two systems contributes independently and gives rise to two different exponents? AIC is a statistical method for distinguishing the best fitting function among multiple candidates. Basically it balances the principles of accuracy (i.e., minimum loss of information) and frugality. Here we show the outcome of four states and human resting-state by AIC (Akaike information criterion)^30^. Respectively, the AIC values calculated for single/double power law are (1) 729564/722132 for healthy humans in resting state, (2) 4016.756 /4020.304 in HS, (3) 1036.135/1025.771 in HW, (4) 2265.964/2266.234 in LW, and (5) 5904.362/5908.362 in LS. Based on this information, we conclude that (1) healthy humans in resting-state for which double power laws fit better, (2) single power law wins out by a small margin for low iso rats. It is worth noting that this result should be treated with cautions because LW and LS rats are hard to remain still during fMRI scanning, (3) high iso WKY rats also favor the double powers, and (4) single power law is a better fit for SHR rats. Recent studies found that SHR (ADHD) children usually exhibit abnormal DMN network^31^. It has also been reported that mental disorder such as Alzheimer^32,33^, depression^34^, Schizophrenia^35^, and ASD^36^ can render DMN abnormal. It remains a pressing task to clarify whether the transition of double powers to single power correlates with abnormal DMN. In summary, power-law distribution can not only reflect the mental condition of our samples, but also reveal detail information about their network properties.

It is desirable to have more samples to maximize our use of Decision Tree to select power law from MRI data of mentally disordered rats. Although our results have demonstrated that the power-law distribution can be analyzed by Decision Tree to classify dosage of ISO and SHR vs. WKY, to vindicate its versatility more promotions are needed, e.g., depression, hypertension, or transient ischemic attack. We have two ideas to improve current understandings of the dynamics of brain: first, establish a relationship between observers (i.e., Decision Tree) and the objects being observed (i.e., power law from different states.). Once this relationship is available, it may function as a starting point to reveal possible connections among different observed objects.

In the TM, several problems remain to be solved: (1) different group samples may exhibit different ISO, age and so on. Although TM has enjoyed success in some transitions, (2) we yet need to determine whether TM is a linear or nonlinear system. Finally, (3) what are the relations between TM and stimuli is also an urgent question.

## Materials and methods

### Process

Step 1: Using Pearson correlation to calculate fMRI (spatial and temporal dimension) to power law distribution. Step 2: Using chi2 algorithm to select important features from degrees and making tree structure during tenfold. Afterwards, outputting the tree with the best ROC.

### Animals: rat

All the experimental animals were admitted by the National Tsing Hua University Institutional Animal Care and Use Committee and complied with experimental guidelines. (https://drive.google.com/file/d/17cHSrbvBxaqpvEb_b-huZahQ7Ti3aqjL/view?usp=sharing).

### Human subjects

We used resting-state fMRI images collected from six normal human subjects to test single and double power law. The datasets were downloaded from the ADHD-200 Consortium (http://fcon_1000.projects.nitrc.org/indi/adhd200). All MRI scans of the datasets were performed in the New York University Child Study Center^37^. The scan protocol was approved by the institutional review boards of the New York University School of Medicine and New York University. The informed consents were obtained from all subjects. The protocols were performed in accordance with HIPAA guidelines and 1000 Functional Connectomes Project protocols. Our retrospective study using the ADHD-200 database was approved by the institutional review boards of National Taiwan University Hospital. The ADHD-200 Consortium provided de-identified datasets are and removed the protected health information.

### Magnetic resonance imaging

Our raw data come from the same source in Ref.^29^, in this section, copyright is owing to the author who wrote “Magnetic resonance imaging” in the Ref.^15^. We scanned all animals with 7-Tesla Bruker Clinscan, which had a volume coil for signal excitation and a brain surface coil for signal receiving. The anesthesia process is operated by 1.4–1.5% isoflurane mixed with O2 at flow rate of 1 L/min. We monitored all rats, made sure the respiratory rate in the range of 65–75 breaths/min while the scanning period, and body temperature maintained at $$37\,^{\circ }\mathrm {C}$$ by a temperature-controlled water circulation machine. During the rs-fMRI experiments, we used gradient echo echo-planar-imaging (EPI) getting the 300 consecutive volumes with 11 coronal slices. The EPI specification is TE/TR $$= 20$$ ms/1000 ms, matrix size is $$64 \times 64$$, FOV$$ = 30 \times 30 \,\,\mathrm{mm}^{2}$$ and slice thickness = 1 mm. We get the anatomical images by turbo-spin-echo (TSE) with scanning parameters of TE/TR$$= 14/4000$$, matrix size = $$256 \times 256$$, FOV$$= 30\times 30\,\, \mathrm{mm}^{2}$$, slice thickness $$= 1 $$mm, number of average = 2. To inspect the result of deep anesthesia, we applied 2.5–2.7% isoflurane mixed with O2, and monitored respiratory rate in the range of 40–45 breaths/min during the whole scanning period.

### Data processing for distribution for degree

Here we analyze our raw data from fMRI Grayscale image. Afterward, we transform them to scalar value matrix by MATLAB 2015a and 2018 version. This matrix is four dimensional, $64 \times 64 \times 11 \times 525$ where the first three components denote spatial position, while the last component refers to the time section in the scanning. Four dimensions render the matrix hard to manipulate, and it costs a lot of computer time. One can use GPU computing to disassemble it to two-dimensional form ($45,056 \times 525$). After using Eq. (1) to calculate all voxels of degree in whole brain, we can get the distribution for degree for any sample. We calculate all 80 rats (each state has 20 samples) in order to obtain the form of the distribution for degree.

### Chi2 algorithm

The feature selection on this study stems from chi2 algorithm^38^ which is designed to discretize numeric attributes based on the $X^{2}$ statistic, and consists of two phases. In the first phase, it begins with a high significance level (sigLevel), Phase 1 is, as a matter of fact, a generalized version of ChiMerge of Kerber. Phase 2 is a ner process of Phase 1. Starting with sigLevel0 determined in Phase 1, each attribute i is associated with a sigLevel[i], and takes turns for merging. Consistency checking is conducted after each at- tribute’s merging. At the end of Phase 2, if an attribute is merged to only one value, it simply means that this attribute is not relevant in representing the original data set. As a result, when discretization ends, feature selection is accomplished.

### Data processing for C4.5 decision tree

In this study, we promote a set of new algorithms to enhance the Identifying effectiveness of SHR and WKY. The classifier algorithms are a combination of chi2 algorithm and C4.5 decision tree (C4.5), the chi2 algorithm evaluates the worth of a subset of attributes and C4.5 speculate the mental disorder. The chi2 algorithm is commonly used for testing relationships between categorical variables. The calculation of the chi2 algorithm is follows $X^{2}= \sum \frac{(f_{0}-f_{e})^{2}}{f_{e}}$, where $f_{0}=$ the observed frequency (the observed counts in the cells) and $f_{e}=$ he expected frequency if NO relationship existed between the variables.The decision tree algorithm is well known for its robustness and learning efficiency with a learning time complexity of $O(n\ log2n)$^39^. C4.5 has been listed in the item 10 algorithms in data mining^40^. It is a popular statistical classifier developed by Ross Quinlan in 1993. Basically, C4.5 is an extension of Quinlan’s earlier ID3 algorithm. In C4.5 the Information Gain split criterion is replaced by an Information Gain Ratio criterion which penalizes variables with many states. C4.5 can be used to generate a decision tree for classification. The learning algorithm applies a divide-and-conquer strategy^41^. to construct the tree. The sets of instances are accompanied by a set of genes (attributes). This classifier has additional features, such as handling missing values, categorizing continuous attributes, pruning decision trees, deriving rules, endotestae Information gain (S, A) of a feature A relative to a collection of examples S, is defined as $Gain(S,A)=Entropy(S)-(\sum \frac{S_V}{S}\times Entropy(S_{V}))$, where Values (A) is the set of all possible values for attribute A, and Sv is the subset of S for which feature A has value v (i.e $S_{V}=\{ s\in S\mid A(s)=v \}$), Note the first term in the equation for Gain is just the entropy of the original collection S and the second term is the expected value of the entropy after S is partitioned using feature A. The expected entropy described by the second term is the direct sum of the entropy of each subset Sv, weighed by the fraction of samples $\frac{\mid S_{V} \mid }{\mid S \mid }$ that belong to $S_{V}$, Gain (S, A) is therefore the expected reduction in entropy caused by knowing the value of feature A. The Entropy is given by $Entropy(S)=\mathop {\sum _{i=1}^{n}} -P_{i}log(P_{i})$.

### Data processing for testing single and double power law

We choose the same method and procedures described in Ref.^41^. Details can be found in Sec.IV^42^.

### Data processing for calculating L

When we calculated the degree for any voxels, the path length (L) between two voxels is defined as the minimum number of links necessary to connect each other. Also, we collect L from data of six rats for each state at the same time. Afterward, MATLAB 2018a is employed to find the max path and average L.

### Data processing for C and structure network patterns

First, we print out all connection information between two voxles for any sample in txt format. Then, insert the data in MATLAB 2018a to obtain the functional brain network pattern. If interested at calculating the clustering coefficient for any voxel linked, you have to get information of degree and the number of links connecting the neighbors. Finally, the average C can be determined from this equation $C=\frac{1}{N\mathop {\sum _{i=1}}C_{i}}$ where *N* is the number of voxels and *i* the voxel number.
